# Case report: C57BL/6NTac and C57BL/6NCrl mice displaying neurological signs after deworming with ivermectin

**DOI:** 10.1177/00236772241286214

**Published:** 2024-12-24

**Authors:** M. Eriksson, S. Nylén

**Affiliations:** 1 Department of Microbiology, Tumor and Cell Biology, Karolinska Institute, Stockholm, Sweden; 2 Department of Microbiology, Swedish Veterinary Agency, Uppsala, Sweden

**Keywords:** Laboratory animal welfare, helminths, rodents, deworming, neurotoxicity

## Abstract

For over 40 years, ivermectin has served as an effective anti-parasitic drug used in human and veterinary medicine. In laboratory animal facilities it is used prophylactically or therapeutically to maintain the health status of the colony or experimentally in studies. Although ivermectin is generally safe to use, there are reports of neurotoxicity associated with ivermectin crossing the blood–brain barrier due to overdosing or blood–brain barrier dysfunction. In mice, P-glycoprotein maintains the blood–brain barrier and mice with a mutation in the P-glycoprotein encoding gene *mdr1a* are 50–100 times more sensitive to ivermectin. Signs of neurotoxicity include ataxia, bradypnea, recumbency, tremor, and death. We report neurotoxicity after ivermectin administration was used for the purpose of eradicating the murine-specific intestinal nematode *Heligmosomoides polygyrus* in C57BL/6NTac and C57BL/6NCrl mice. The mice were dewormed by subcutaneous administration of 10 or 20 mg/kg ivermectin to eradicate all stages of *Heligmosomoides polygyrus*. At 24–48h after deworming, 5% (*n* = 4) of the mice presented with tremor, ataxia, and/or head tilt. The affected mice were euthanised and gross pathological findings were found in one of the four mice (left-sided hydronephrosis). We assume that the observed neurological effects were due to defects in the blood–brain barrier, overdosing or individual sensitivity. This report provides a reason for caution when deworming laboratory mice subcutaneously with ivermectin at doses of 10 mg/kg or higher.

## Rapport d’expérience: Souris C57BL/6NTac et C57BL/6NCrl présentant des signes neurologiques après traitement vermifuge à l'ivermectine Résumé

L'ivermectine est un médicament antiparasitaire efficace utilisé depuis plus de 40 ans en médecine humaine et vétérinaire. Elle est utilisée à titre prophylactique ou thérapeutique dans les installations pour animaux de laboratoire afin de maintenir l'état de santé de la colonie ou dans le cadre d’études expérimentales. Bien que l’utilisation de l'ivermectine soit généralement sans danger, il existe des rapports de neurotoxicité associée au franchissement de la barrière hémato-encéphalique par l'ivermectine en raison d'un surdosage ou d'un dysfonctionnement de cette barrière. Chez la souris, la glycoprotéine P maintient la barrière hémato-encéphalique et les souris présentant une mutation du gène codant pour la glycoprotéine P *mdr1a* sont 50 à 100 fois plus sensibles à l’ivermectine. Les signes de neurotoxicité comprennent l'ataxie, la bradypnée, la position couchée, les tremblements et la mort. Nous rapportons une neurotoxicité après administration d'ivermectine dans le but d'éradiquer le nématode intestinal spécifique de la souris *Heligmosomoides polygyrus* chez les souris C57BL/6NTac et C57BL/6NCrl. Les souris ont fait l’objet d’un traitement vermifuge par administration sous-cutanée de 10 ou 20 mg/kg d'ivermectine pour éradiquer tous les stades d'*Heligmosomoides polygyrus*. 24 à 48 heures après l’administration du vermifuge, 5% (n = 4) des souris présentaient des tremblements, une ataxie et/ou une inclinaison de la tête. Les souris affectées ont été euthanasiées et des signes pathologiques généraux ont été trouvés chez l'une des quatre souris (hydronéphrose gauche). Nous supposons que les effets neurologiques observés étaient dus à des défauts de la barrière hémato-encéphalique, à un surdosage ou à une sensibilité individuelle. Ce rapport justifie la prudence lors du traitement vermifuge des souris de laboratoire par voie sous-cutanée avec de l'ivermectine à des doses de 10 mg/kg ou plus.

## Fallbericht: C57BL/6NTac- und C57BL/6NCrl-Mäuse mit neurologischen Symptomen nach Entwurmung mit Ivermectin Abstract

Seit über 40 Jahren ist Ivermectin ein wirksames Antiparasitikum, das in der Human- und Veterinärmedizin eingesetzt wird. In der Versuchstierhaltung dient es prophylaktisch oder therapeutisch zur Gewährleistung des Gesundheitszustands der Kolonie oder zu Versuchen in Studien. Obgleich Ivermectin im Allgemeinen sicher in der Anwendung ist, gibt es Berichte über Neurotoxizität im Zusammenhang mit dem Überschreiten der Blut-Hirn-Schranke durch Ivermectin aufgrund einer Überdosierung oder einer Störung der Blut-Hirn-Schranke. Bei Mäusen wird die Blut-Hirn-Schranke durch das P-Glykoprotein aufrechterhalten, und Mäuse mit einer Mutation im P-Glykoprotein-kodierenden Gen *mdr1a* sind 50 bis 100 Mal empfindlicher gegenüber Ivermectin. Zu den Anzeichen von Neurotoxizität gehören Ataxie, Bradypnoe, Rückenlage, Tremor und Tod. Wir berichten über Neurotoxizität nach Verabreichung von Ivermectin zur Eradikation des mäusespezifischen Darmnematoden *Heligmosomoides polygyrus* bei C57BL/6NTac- und C57BL/6NCrl-Mäusen. Die Mäuse wurden durch subkutane Verabreichung von 10 bzw. 20 mg/kg Ivermectin entwurmt, um alle Stadien von *Heligmosomoides polygyrus* zu beseitigen. 24 bis 48 Stunden nach der Entwurmung traten bei 5% (n = 4) der Mäuse Tremor, Ataxie und/oder Kopfneigung auf. Die betroffenen Mäuse wurden euthanasiert und bei einer der vier Mäuse wurde ein schwerwiegender pathologischer Befund festgestellt (linksseitige Hydronephrose). Wir gehen davon aus, dass die beobachteten neurologischen Effekte auf Defekte der Blut-Hirn-Schranke, Überdosierung oder individuelle Empfindlichkeit zurückzuführen sind. Dieser Bericht gibt Anlass zur Vorsicht bei der subkutanen Entwurmung von Labormäusen mit Ivermectin in Dosen von 10 mg/kg oder höher.

## Informe de caso: Ratones C57BL/6NTac y C57BL/6NCrl con signos neurológicos tras la desparasitación con ivermectina Resumen

Durante más de 40 años, la ivermectina ha sido un eficaz fármaco antiparasitario utilizado tanto en la medicina humana como en la veterinaria. En los centros de animales de laboratorio se utiliza profiláctica o terapéuticamente para mantener el estado de salud de la colonia o experimentalmente en estudios. Aunque la ivermectina es generalmente segura de usar, existen informes de neurotoxicidad asociados con el cruce de la barrera hematoencefálica por parte de la ivermectina debido a una sobredosis o a una disfunción de la barrera hematoencefálica. En los ratones, la glicoproteína P mantiene la barrera hematoencefálica y los ratones con mutación en el gen *mdr1a* que codifica la glicoproteína P son entre 50 y 100 veces más sensibles a la ivermectina. Los signos de neurotoxicidad incluyen ataxia, bradipnea, decúbito, temblores y la muerte. Informamos de la neurotoxicidad tras la administración de ivermectina para erradicar el nematodo intestinal *Heligmosomoides polygyrus*– que afecta específicamente a murinos– en ratones C57BL/6NTac y C57BL/6NCrl. Los ratones fueron desparasitados mediante la administración subcutánea de 10 o 20 mg/kg de ivermectina para erradicar todas las fases del *Heligmosomoides polygyrus*. A las 24 - 48 horas después de la desparasitación, el 5% (n = 4) de los ratones presentaron temblor, ataxia y/o inclinación de la cabeza. Los ratones afectados fueron sacrificados y se encontraron hallazgos patológicos macroscópicos en uno de los cuatro ratones (hidronefrosis del lado izquierdo). Suponemos que los efectos neurológicos observados se debieron a defectos en la barrera hematoencefálica, sobredosis o sensibilidad individual. Este informe advierte sobre la necesidad de precaución al desparasitar ratones de laboratorio por vía subcutánea con ivermectina con dosis de 10 mg/kg o superiores.

## Introduction

Ivermectin, a dihydro-derivate of avermectin produced by *Streptomyces avermitilis*, was discovered during the 1970s in Japan by the Kitasato Institute in collaboration with Merck, Sharp and Dohme.^1^ It was first approved for veterinary use in 1981 and then for human use in 1988.^1^ It is effective against a broad range of both endo- and ectoparasites including *Strongyloides*, *Trichuris trichiura,* lungworms, and fur mites (*Myobia musculi* and *Mycoptes musculinus*).^2–5^ The drug exerts its effect by binding to glutamate-gated chloride channels in neurons and the muscle cells of invertebrates.^6^ The binding results in hyperpolarisation of the cells causing neuromuscular paralysis and in some parasites death. Ivermectin can also bind to GABA-gated chloride channels, but as the drug does not readily cross the blood–brain barrier at recommended doses, the risk of toxicity in mammals has been deemed almost negligible.^1,7,8^ Nevertheless, toxic effects have been reported including ataxia, coma, gastrointestinal symptoms, and confusion after overdosing in humans or recommended dosing in individuals with nonsense mutations in the *ABCB1*/*mdr1a* gene.^9–12^ The therapeutic doses according to the manufacturer’s instruction for human and veterinary preparations are 0.15–0.5 mg/kg.^13,14^ The acute oral toxicity LD_50_ dose in mice is 25 mg/kg and 50 mg/kg in rats as defined in the safety data sheet of ivermectin produced by Merck. Doses ranging from 0.0002% in the diet to 20 mg/kg have proven to be well tolerated by mice.^5,15–19,23^ Nevertheless, 10 mg/kg administered intraperitoneally resulted in 14% mortality in one study.^17^ In addition, effects on some behaviours have been observed in AKR/J, C57BL/6 and 129/SvEv mice after 8 weeks of administration of 0.008 mg/ml 0.08% Ivomec® sheep-drench in drinking water.^20^

Neurological signs after ivermectin administration result from interaction of the drug with GABA receptors in the central nervous system after breaching the blood–brain barrier.^21^ The *mdr1a* P-glycoprotein is essential for maintaining an intact blood–brain barrier in mice.^22^ Mice with a homozygous mutation in the *mdr1a* gene have increased drug levels in several organs but in particular in the brain which, together with impaired drug elimination, results in a 50–100 times higher sensitivity to ivermectin.^16,18^ Mice showing signs of ivermectin-induced neurotoxicity present with ataxia, recumbency, bradypnea, tremor, and ultimately death.^16^ In addition, administration of P-glycoprotein-modulating drugs (e.g. cyclosporin) leads to an accumulation of ivermectin in the brain with subsequent neurological symptoms in mice.^17^

Here we report symptoms of neurotoxicity in C57BL/6NTac and C57BL/6NCrl mice after subcutaneous administration of 10 or 20 mg/kg ivermectin to eradicate the murine-specific intestinal nematode *Heligmosomoides polygyrus* (HP). The mice were enrolled in separate studies in a project investigating the effects of HP on influenza virus immunity. The ivermectin doses were selected based on published data of successful elimination of HP without reported adverse effects.^23^ The mice were healthy upon visual inspection on the day of ivermectin administration. One to two days after treatment, four out of 80 mice from two separate experiments had to be euthanised due to ataxia and tremor.

## Method

### Ethics statement

The project was performed according to EU Directive 2010/63/EU on the protection of animals used for scientific purposes. Protocols for animal experiments were approved by the Central Ethical Committee on Animal Experiments in Sweden (Approval no. 2019-067) and the Regional Animal Ethics Committee of Stockholm, Sweden (Approval no. 6738-2019). Humane endpoint criteria were defined in the ethical permit and the mice were assessed using a scoring sheet for mice by Karolinska Institute.

### Housing of mice

All mice were housed in conventional, open-top cages with an 820 cm^2^ floor area with aspen bedding (Tapvei, Harjumaa, Estonia) and had *ad libitum* access to water and Mouse breeding V1124-300 feed (ssniff Spezialdiäten GmbH, Soest, Germany) at the animal facility of the Swedish Veterinary Agency, Uppsala, Sweden. The following enrichment was used: polycarbonate mouse tent (Datesand, Stockport, UK), cellulose paper, 1–2 sunflower seeds (LBS Biotechnology, Horsham, UK) per mouse and day, and hay (local farmer, Uppsala, Sweden) autoclaved at the animal facility.

### Statistical analysis

GraphPad Prism version 10.1.2 (GraphPad Software, Boston, Massachusetts, USA) was used for statistical analysis. A mixed-effects analysis with Geisser–Greenhouse correction for variability and the Šidák test for multiple comparisons were used for comparison of body weight changes between the groups at different timepoints. The following was used to denote statistical significance: *p < 0.05, **p < 0.01, ***p < 0.001.

## Report

This report describes the unexpected findings after the deworming of mice that were part of a project that aimed to study immunity during co-infection with the murine-specific, intestinal nematode *HP* (formerly *Nematospiroides dubius)*, and the influenza A virus. HP infection in mice is used to model human gastrointestinal helminth infection and allows studies of the immunomodulatory effects a parasite infection induces *in vivo*. The experimental design for each group is presented in Table 1. All studies in the project were open label. The group size was based on previous data. Fewer mice were used if the group or experiment was a pilot within the aforementioned project. As part of the project, the mice were dewormed to eradicate the HP infection. To control for the effects of deworming, ivermectin was also given to animals not infected by worms. All mice used were healthy upon clinical examination on the day of deworming with 10–20 mg/ml Ivomec® vet. (Boehringer Ingelheim Animal Health, Ingelheim am Rhein, Germany) diluted in sterile phosphate buffered saline administered subcutaneously (23-gauge needle, volume up 300 µl). Based on our own experience using benzimidazoles, praziquantel and pyrantel pamoate, HP is difficult to eradicate. Thus, for the purpose of the project where eradication was deemed important, we opted for the drug and a dose described to achieve this. The dose in Case 1 was chosen based on work by Wahid et al. where they achieved total eradication of all HP developmental stages with 20 mg/kg of ivermectin diluted in distilled water administered subcutaneously, without adverse effects observed in the mice.^23^ In Case 2, 10 or 20 mg/kg ivermectin was used since we observed neurological symptoms in two mice in Case 1. It should be noted that in Case 2, one mouse displaying neurological symptoms was a control with no previous procedure and the other mouse had undergone different procedures at various timepoints prior to deworming with ivermectin compared to the mice that displayed symptoms in Case 1. Thus, there was no obvious correlation between previous treatments and the symptoms induced after ivermectin administration.

**Table 1.** Description of experimental procedures the mice in Groups 1–9 had undergone before deworming at Day 0 (D0). Weight (g) indicates individual weights recorded at two days prior to deworming for Groups 1–4 and at D0 for Groups 5–9. Maximum (max) and minimum (min) weights recorded up to Day 32 after deworming are indicated in the table.

Case 1 C57BL/6NT ac	Group	−5 months	−54 days	−53 days	−6 to −12 wks	− 38 days	−17 days	−12 days	Ivermectin (D0)	Animal^a^	Weight (g)	Max	Min		Neurological symptoms and necropsy findings
	1									1a	28.8	33.5	28.8		
										1b	28.4	31.5	28.4		
										1c	28.4	31.6	28.4		
										1d	28.4	33.8	28.4		
										1e	27.6	31.3	27.6		
										1f	27.8	31.6	27.8		
										1g	27.7	32	27.7		
										1h	29.1	32.6	29.1		
										1i	27.3	30	27.3		
										1j	28.4	31.5	28.4		
	2					PR8				2a	25.5	28	25.5		
										2b	24	27.2	24		
										2c	22.8	25.2	22.8		
										2d	27.4	30.6	27.4		
										2e	28	31.4	28		
										2f	27	31.6	27		
										2g	27.2	30	27.2		
										2h	28.9	31.7	28.9		
										2i	27.7	31.1	27.7		
										2j	28.6	33.2	28.6		
										2k	27.2	30.6	27.2		
										2l	28.5	31.6	28.5		
										2m	26.5	30	26.5		
										2n	27.3	32.1	27.3		
										2o	27.3	30	27.3		
	3						HP		20 mg/kg	3a	26.4	30	26.4		
										3b	25.6	27.9	25.6		
										3c	23.8	25.7	23.4		
										3d	28.9	30.8	28.9		
										3e	27	30.1	27		
										3f	27.8	29.8	27.7		
										3g	27.6	27.6	27.6		D2: Ataxia, tremor and moderate piloerection. Moribound.
										3h	27.4	29	27.4		
										3i	27.9	30.2	27.9		
										3j	29.2	29.2	29.2		D2: Ataxia, tremor and moderate piloerection.
	4					PR8	HP		20 mg /kg	4a	24	25.9	24		
										4b	28.6	31.7	28.6		
										4c	27.1	28	27.1		
										4d	27.4	30	26.4		
										4e	28	30.7	28		
										4f	27.4	30	27.4		
										4g	28.4	31	28.4		
										4h	28.5	32	28.5		
										4i	27	30.5	27		
										4j	29.2	32.9	29.2		
										4k	27.1	30.8	27.1		
										3l	25.9	28.9	25.9		
										4m	22.8	25	22.5		
										4n	27.3	30.5	26.7		
										4o	27.1	30.3	27.1		
Case 2 C57BL/6N Crl	Group	−5 months	−54 days	−53 days	−6 to −12 wks	−38 days	−17 days	−12 days	Ivermectin (D0)	Animal^a^	Weight (g)	max	min	Piloerection	Neurological symptoms and necropsy findings
	5				HP				20 mg/kg	5a	31.8	32	30.8	Yes	
									10 mg/kg	5b	32.6	32.7	31.8	Yes	
									10 mg/kg	5c	31.8	31.8	30.2	Yes	
									10 mg/kg	5d	32.4	32.4	31	Yes	
									20 mg/kg	5e	29.7	29.7	28.7	Yes	
									10 mg/kg	5f	22.1	27.5	22.1	No	
									20 mg/kg	5g	30.1	30.3	28.6	Yes	
									20 mg/kg	5h	26.8	26.8	26.3	Yes	
									20 mg/kg	5i	26.1	27.1	25.8	Yes	
									10 mg/kg	5j	27.9	28.3	27.8	No	
									10 mg/kg	5k	28	28.7	27.9	No	
									10 mg/kg	5l	27	27	25.2	Yes	
									20 mg/kg	5m	30.2	30.4	29	Yes	
									20 mg/kg	5n	33.5	33.5	32	Yes	
									10 mg/kg	5o	32.9	32.9	31.8	Yes	
									10 mg/kg	5p	34.9	34.9	32.5	Yes	
									10 mg/kg	5q	29	30	28.3	Yes	
									20 mg/kg	5r	32.5	32.6	30.8	Yes	
	6		PR8					PR8	10 mg/kg	6a	28.1	28.4	27.1	Yes	
	7			IgY				PR8	20 mg/kg	7a	28.3	28.3	26.2	Yes	D1: Moderate ataxia, resting tremor, mild left-sided head-tilt.
			PR8+IgY						20 mg/kg	7b (F)	22.5	22.5	20.5	Yes	
									10 mg/kg	7c (F)	21.7	22.6	21.7	No	
	8	Vaxigrip tetra				HP			20 mg/kg	8a	33	33.5	31.9	Yes	
									10 mg/kg	8b	33.5	33.5	31.8	Yes	
									20 mg/kg	8c	33.9	33.9	31	Yes	
									20 mg/kg	8d	34.4	34.4	30.9	Yes	
	9								10 mg/kg	9a	16.4	19.1	16.1	Yes	
									10 mg/kg	9b (F)	15.5	15.5	14.2	Yes	
									20 mg/kg	9c (F)	16.5	17.6	15.6	Yes	D1: Moderate ataxia, resting tremor, mild left-sided head-tilt. Left-sided hydronephrosis.
									20 mg/kg	9d (F)	13.9	17	13.6	Yes	

*Note*: PR8: intranasal infection with 0.75–2 × 10^3^ TCID_50_ influenza virus (strain A/Puerto Rico/8/1934 H1N1); HP: oral infection with 200–300 third-stage *Heligmosomoides polygyrus* larvae; IgY: antiviral treatment with influenza virus-specific IgY; (F): female.

a Cage is indicated by bold lines.

b ‘Yes’ in column ‘Piloerection’ indicates that piloerection was observed at any timepoint up to 12 days after deworming in Case 2.

### Case 1

**Figure 1.**
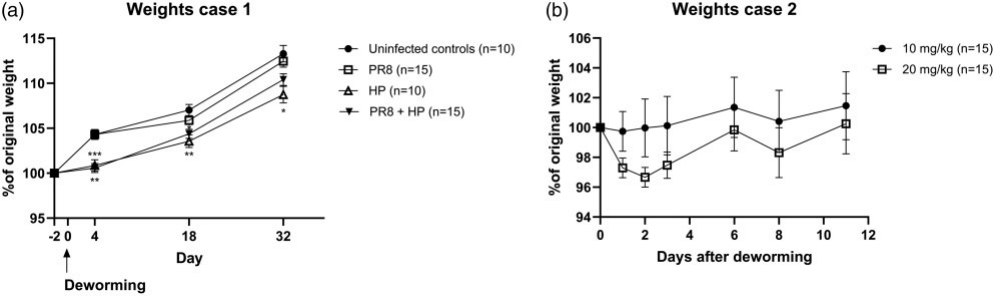
Deworming with ivermectin induces weight loss or reduced weight gain in growing mice. Recorded weights of mice belonging to Case 1 (a). The weights of the mice were recorded at predetermined timepoints before and after deworming. At each timepoint the group means were compared with the uninfected controls. Weights after administration of 10 mg/kg or 20 mg/kg ivermectin subcutaneously (b). All mice in Case 2 are included in the graph. Data are shown as mean ± standard error of the mean. PR8: intranasal infection with 0.75–2 × 10^3^ TCID_50_ influenza virus (strain A/Puerto Rico/8/1934 H1N1); HP: oral infection with 200–300 third-stage *Heligmosomoides polygyrus* larvae.

Fifty 3- to 4-week-old, 16.0–22.0 g, male murine pathogen-free (MPF) C57BL/6NTac (Taconic Biosciences, Ejby, Denmark) were randomly assigned to Groups 1–4 upon arrival, by a technician who placed the mice in different cages where each cage was an experimental unit. Male mice were chosen due to their delayed expulsion of HP compared to females. HP-infected groups were dewormed with one single, subcutaneous injection of 20 mg/kg Ivomec vet 17 days after infection with HP. Two mice in Group 4, housed in the same cage, exhibited severe ataxia, tremor and moderate piloerection 2 days after deworming. One of the mice was moribund upon clinical examination and the other mouse made attempts to move in the cage. Both mice were euthanised, there were no gross pathological findings. Most of the remaining dewormed mice displayed mild piloerection but no neurological symptoms. For up to 32 days after deworming, the dewormed mice had a decreased weight gain with a less steep weight gain curve than the mice that were not dewormed (Figure 1(a)).

### Case 2

In another set of experiments, five females aged 4–10 weeks (13.9–22.1 g) and 25 males aged 4–20 weeks (16.4–41.3 g), all specific-pathogen-free (SPF) C57BL/6NCrl bred inhouse as second or third generations, were dewormed with either 10 mg/kg (*n* = 15) or 20 mg/kg (*n* = 15) Ivomec vet, administered subcutaneously. We used two different doses due to the neurological symptoms we observed in two mice in Case 1. Mechanical randomisation was used within each cage (i.e. block) for determination of ivermectin dose. Health monitoring at the facility was performed according to FELASA recommendations using sentinels.^24^ The following agents were considered enzootic at the facility: murine norovirus, *Helicobacter spp*., and pasteurellaceae.

One day after deworming, one female from Group 9 aged 30 days and one male aged 11 weeks from Group 7 dewormed with 10 mg/kg and 20 mg/kg respectively, displayed neurological symptoms and had lost 8.4% (1.2 g) and 7.5% (2.1 g) weight respectively. The mice presented with moderate ataxia, general resting tremor with more pronounced symptoms from the tail, and mild left-sided head-tilt. Both mice were euthanised and necropsied. The female had left-sided hydronephrosis;the male had no gross pathological findings. Overall, five mice dewormed with 10 mg/kg and 11 mice dewormed with 20 mg/kg had up to 8.4% weight loss during the first 2 days after deworming. Mice dewormed with 20 mg/kg had a trend towards an increased weight loss compared to mice dewormed with 10 mg/kg (Figure 1(b)).

## Discussion

In this case report, neurological signs in mice induced by subcutaneous administration of ivermectin are described. The mice had undergone different procedures or no procedure prior to deworming; thus, we attribute the observed adverse effects to ivermectin. We cannot exclude that the previous procedures, or the younger age of and lower starting weight in the previously untreated group (Case 2 group), affected the ability to tolerate ivermectin, but neither do we have any evidence to support such a suggestion.

Due to its broad effects, ivermectin is a commonly used anti-parasitic drug and an important tool for eradication of susceptible parasites in laboratory animal facilities. However, as demonstrated in this case report, the choice of dose is important since higher doses may cause adverse effects in clinically healthy animals.

Weakness in the blood–brain barrier is required for ivermectin to access the central nervous system. This is achieved by the inability of P-glycoprotein to maintain the blood–brain barrier as a consequence of either ivermectin overdosing, and/or P-glycoprotein dysfunction or inhibition. The neurological signs our mice exhibited could be either due to overdosing, increased individual susceptibility or a defect in the blood–brain barrier. CF-1 mice have a spontaneous mutation in the *mdr1a* gene resulting in P-glycoprotein deficiency in the intestine, blood–brain barrier, and placenta.^22,25^ To the best of our knowledge, no such mutation has been described in C57BL/6 mice. Of note, both mice in Case 2 that displayed neurological symptoms came from the same breeding couple but from two different litters. Although housed in the same cage, the ancestry of the mice in Case 1 is unknown as they were supplied by a vendor. Therefore, we cannot exclude, nor confirm, a congenital defect in the blood–brain barrier in the mice.

Of the four mice in this report, macroscopic lesions were only observed in one mouse at necropsy. However, the impact of the finding is uncertain as ivermectin is mainly excreted in an unaltered form in faeces whereas renal excretion only accounts for 1%–2%.^26–28^ Hence, reduced renal function should not result in accumulation of the drug in such concentrations that would enable crossing of the blood–brain barrier. Nevertheless, the presence of one congenital malformation does not exclude additional malformations, including defects in the blood–brain barrier.

Reported LD_50_ doses in mice vary substantially and range from 14–15 mg/kg after intraperitoneal injection to 50–60 mg/kg after oral administration.^16,17^ Differences in LD_50_ and tolerated doses might be due to the use of different administration routes and strains. Ivermectin is highly lipophilic and drug availability depends largely on the route of administration, with subcutaneous surpassing intravenous and oral administration.^29^ There is a possibility that overdosing is easier to achieve with subcutaneous administration.

## Conclusion

Ivermectin is an important anti-parasitic drug that in a laboratory animal setting can be used to deworm mice as part of a study, as a prophylaxis in quarantined animals or therapeutically in case of a parasite outbreak in a facility. This report exemplifies that the use of doses of 10 and 20 mg/kg administered subcutaneously can induce neurological symptoms (e.g. ataxia) in some mice. The severity of the symptoms might call for euthanasia for animal welfare reasons and therefore care should be taken when choosing the dose and route of administration when deworming mice with ivermectin.

## Data Availability

The data in the case report can be made available by the authors upon request.
